# The Symmetry of Viral Sialic Acid Binding Sites—Implications for Antiviral Strategies

**DOI:** 10.3390/v11100947

**Published:** 2019-10-14

**Authors:** Nils H. Rustmeier, Michael Strebl, Thilo Stehle

**Affiliations:** 1 Interfaculty Institute of Biochemistry, University of Tuebingen, 72076 Tuebingen, Baden-Wuerttemberg, Germany; nils.rustmeier@uni-tuebingen.de (N.H.R.); michael.strebl@uni-tuebingen.de (M.S.); 2 Department of Pediatrics, Vanderbilt University School of Medicine, Nashville, TN 37232, USA

**Keywords:** sialic acid, non-enveloped viruses, antiviral compounds, multivalency, inhibition

## Abstract

Virus infections are initiated by the attachment of the viral particle to protein or carbohydrate receptors on the host cell. Sialic acid-bearing glycan structures are prominently displayed at the cell surface, and, consequently, these structures can function as receptors for a large number of diverse viruses. Structural biology research has helped to establish the molecular bases for many virus–sialic acid interactions. Due to the icosahedral 532 point group symmetry that underlies many viral capsids, the receptor binding sites are frequently arranged in a highly symmetric fashion and linked by five-fold, three-fold, or two-fold rotation axes. For the inhibition of viral attachment, one emerging strategy is based on developing multivalent sialic acid-based inhibitors that can simultaneously engage several of these binding sites, thus binding viral capsids with high avidity. In this review, we will evaluate the structures of non-enveloped virus capsid proteins bound to sialylated glycan receptors and discuss the potential of these structures for the development of potent antiviral attachment inhibitors.

## 1. Introduction

The cell membranes of eukaryotes are decorated with a large number of chemically and structurally diverse carbohydrates. These so-called glycans form a protective layer at the interface between a cell and its environment. Components of this layer are synthesized and assembled by a large set of enzymes that differ among species, thus helping to provide host-specific glycan structures.

**Figure 1:**
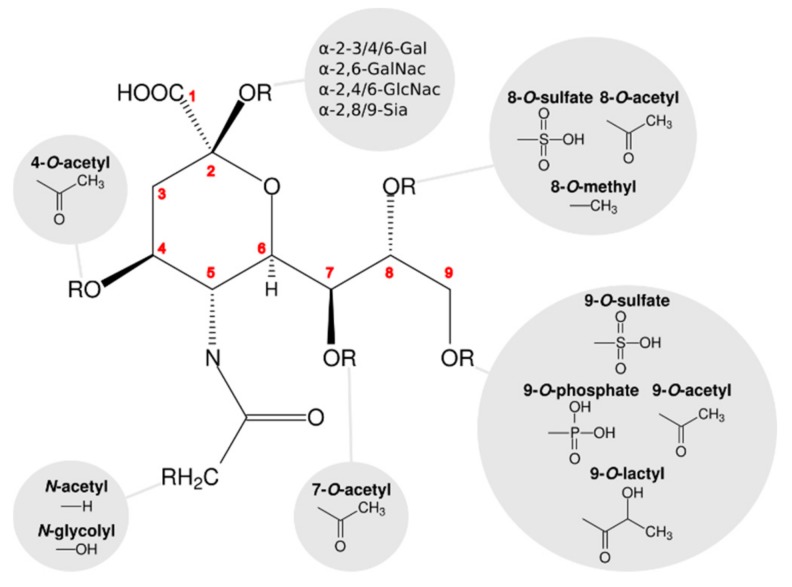
Schematic of sialic acid variants. The *N*-acetylneuraminic acid (Neu5Ac) scaffold provides the chemical basis for numerous naturally occurring modifications in sialic acids.

Despite some differences, the carbohydrate building blocks of the glycan layer (monosaccharides) are largely identical in all cases. Commonly found monosaccharides in glycan structures are glucose (Glc), galactose (Gal), their N-acetylated forms *N*-acetylglucosamine (GlcNAc) and *N*-acetylgalactosamine (GalNAc), and mannose (Man). These building blocks constitute the bulk of most glycans. However, two particularly important sugar classes are missing here: fucoses and sialic acids. While Glc(-NAc) and Gal(-NAc) are usually components of the scaffold, fucose (Fuc) and sialic acids are often added as head groups to a glycan. While fucose is, for example, an important determinant in histo-blood group antigens (HBGA), sialic acids are major components of the glycan portions of gangliosides and the glycan structures of many membranous proteins in eukaryotes. Sialic acids are based on a nine-carbon acidic α-keto sugar framework (Figure 1). Due to their anionic nature, sialic acids contribute to the negative net charge of the cell surface. Sialic acids are abundant in the animal kingdom and are found in different forms. Their most common form is *N*-acetylneuraminic acid (Neu5Ac), which also constitutes the chemical basis for other sialic acids. The hydroxylation of the N-acetyl group of Neu5Ac gives rise to *N*-glycolylneuraminic acid (Neu5Gc), another commonly found sialic acid in animals. While Neu5Gc cannot be synthesized in humans due to a gene defect, humans can acquire this sialic acid through their diet [1]. Additional chemical modifications of Neu5Ac can be introduced at its four hydroxyl groups (O4, O7, O8, and O9), including O-acetylation, O-sulfation, O-methylation, O-lactylation, or O-phosphorylation. In total, over 50 different naturally-occurring sialic acids have been identified [2,3,4]. Sialic acids in glycans occur as the α-anomer and are linked via α-2,3 and α-2,6 glycosidic bonds to scaffold Gal(NAc) or GlcNAc moieties, respectively, or via α-2,8 or α-2,9 glycosidic bonds to other sialic acids. The type and distribution of sialic acids, and their connection to the remaining glycan structure, are highly specific for different tissues and host species. Examples of differences between tissues in the same host can be found in human airways and eyes, where sialic acid is usually linked to other sugars via α-2,6 glycosidic and α-2,3 glycosidic bonds, respectively. In birds, which are important habitats for many viruses, the α-2,3 linkage of sialic acid prevails [5]. However, it should be noted that the type of glycosidic linkage is only one of several determinants of virus and host tropism.

Glycans terminating in sialic acid are prominently expressed at the cell surface. They are, therefore, often easily accessible and serve as the initial contact points for many viruses in different families [6,7]. While some of these glycans function as attachment receptors to simply tether a virus to the target cell membrane, others act as entry receptors and mediate binding of the virus, as well as the delivery of the viral genome into the cytoplasm. In the latter case, the attachment step is often followed by the recruitment of secondary (co-)receptors and endocytosis factors, eventually leading to cell entry of the virus particle or its components and infection of the cell. Among the enveloped viruses that recognize sialic acid-containing receptors are members of the families *Coronaviridae*, *Paramyxoviridae* and *Orthomyxoviridae* [8,9,10,11,12]. In non-enveloped viruses, sialic acid-containing glycans serve as attachment receptors for members of the *Parvoviridae*, *Picornaviridae*, *Caliciviridae*, *Polyomaviridae*, *Reoviridiae*, and *Adenoviridae* [13,14,15,16,17,18,19,20,21,22,23,24,25,26]. Structural biology has provided precise views of how these pathogens interact with sialylated glycans, and although the binding modes differ among the viruses listed above, several common principles have emerged. (i) The viral binding sites for sialylated glycans are typically surface-exposed and feature a small number of contacts. The affinities of the interactions are, therefore, quite low (in the millimolar range) [27,28,29,30]. Firm adhesion of the virus to the cell surface is achieved through the engagement of multiple receptors via identical binding sites, which is known as avidity. (ii) In all cases investigated to date, the sialic acid itself mediates the majority of contacts with the viral capsid, with a smaller number of additional contacts formed to neighboring monosaccharides. (iii) Most viruses are highly specific in the context in which sialic acid is presented; that is, they only recognize sialylated glycans featuring, for example, α-2,3-linked sialic acid but do not engage sialylated glycans carrying α-2,6-linked or α-2,8-linked sialic acid. (iv) Although the database remains small, some viruses can discriminate between the many different modifications of sialic acids, and, as some of these modifications, are species-specific, this phenomenon can contribute to the ability of a virus to only infect species that express a particular sialic acid modification.

The available structural information on virus–receptor interactions is crucial to enable the rational design of therapeutic compounds. Due to the surface-exposed binding mode and the weak individual interactions between sialic acids and their cognate virus proteins, modifying sialic acid to achieve high-affinity binding is challenging. However, viruses possess many identical binding sites that are often linked by symmetry operators, and thus multivalent and symmetric ligands that target several binding sites could result in high-affinity interactions.

**Figure 2:**
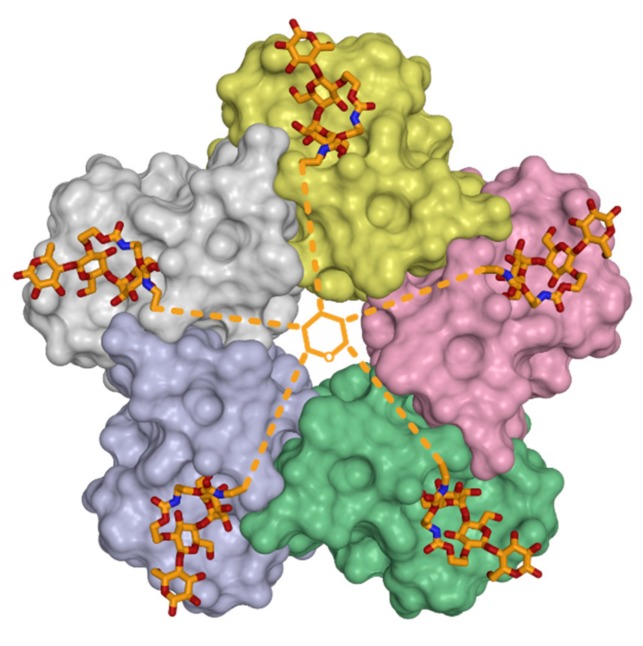
An example of a tailored multivalent inhibitor. The globotriaosylceramide-binding B-subunit of Shiga-like toxin (SLT) forms pentamers and serves as target for the pentavalent inhibitory compound STARFISH, which has been functionalized with the p^k^ trisaccharide. The STARFISH compound exploits the symmetric structure of its target and binds to SLT with a subnanomolar affinity [34]. The SLT pentamer is shown as a protein surface with single protomers colored in grey, yellow, pink, green, and light blue, respectively. The STARFISH compound is shown in stick representation with carbon, nitrogen and oxygen atoms colored in orange, dark blue and red, respectively. Missing parts of the scaffold structure are schematically indicated as orange lines (PDB ID 1qnu). All protein representations in the figures of this review were generated using PyMOL (Schrödinger Inc.).

The strategy of employing a carbohydrate-based, multivalent, and symmetric inhibitor that matches the symmetry of the binding sites in a multimeric target protein was first applied in the context of the bacterial Shiga-like toxin (SLT). SLT consists of an enzymatic domain A and a pentameric, cell-binding domain B [31]. The crystal structures revealed that the B domain pentamer recognizes the p^k^ trisaccharide portion (αGal1-4βGal1-4βGlc) of its physiologic ganglioside receptor, globotriaosylceramide (Gb_3_) [32,33]. In order to achieve high affinity binding, Kitov et al. [34] designed the STARFISH compound, a quasi-symmetric, pentavalent molecule with a central glucose motive carrying five linkers that terminate in dimeric p^k^ trisaccharides (Figure 2). X-ray crystallography of the toxin-inhibitor complex revealed a sandwich-like arrangement of two SLT B-pentamers intercepted by one STARFISH molecule. All five B-pentamer binding sites were simultaneously occupied by the inhibitor. In line with this, affinity measurements showed an increase in the inhibition potency from a millimolar affinity for the monovalent receptor (p^k^ trisaccharide) to a subnanomolar affinity for the STARFISH compound. This concept of targeting multiple, symmetric receptor binding sites by multivalent inhibitors is also applicable for many viruses, since viral capsids are often icosahedral and, therefore, highly symmetric structures.

## 2. Symmetry in Virus Structures and Their Sialic Acid Binding Sites

In this chapter, we will introduce some universal concepts of virus capsid geometry and architecture, focusing in particular on non-enveloped viruses that bind sialic acid-based receptors. We will highlight the local symmetries that relate the sialic acid binding sites in different viral attachment proteins to each other. These local symmetries can serve as a useful framework for the rational design of multivalent virus-targeting inhibitors, similar to the approach used to develop the STARFISH compound. This strategy has been successfully applied to several viruses, as we will show in chapter 3. However, in order to evaluate this approach, we first need to introduce the symmetry elements that guide viral capsid assembly.

**Figure 3:**
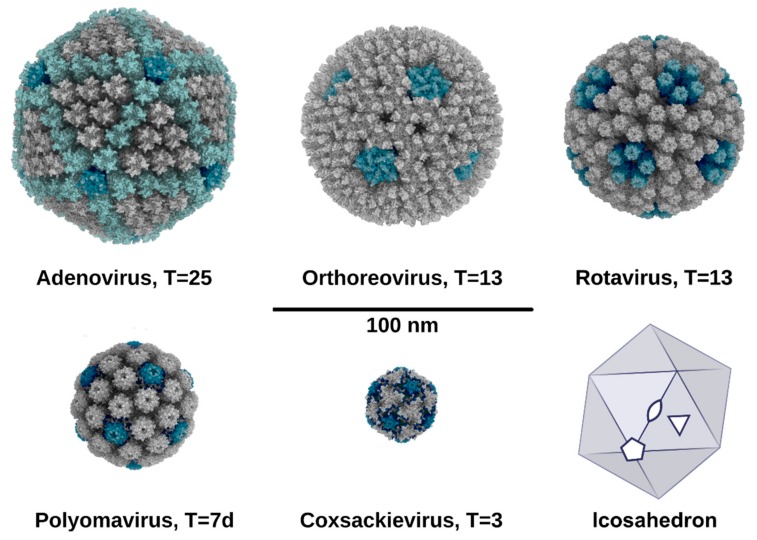
Representation of the architecture and symmetry of a selection of non-enveloped, sialic acid binding viruses. Particle illustrations are sorted by descending virion diameter, disregarding any protruding domains or fibers. The views are positioned along the icosahedral two-fold axes. Five-fold vertices and the associated capsid proteins are highlighted in blue. A schematic view of an icosahedron in the same orientation is shown on the lower right, with two-fold, three-fold and five-fold axes indicated as ellipse, triangle and pentagon, respectively. PDB IDs 6b1t (adenovirus), 2cse (orthoreovirus), 3kz4 (rotavirus), 1sid (polyomavirus), and 4q4w (coxsackievirus).

Typically, small viral genomes can only encode a low number of structural capsid proteins. These capsid proteins often form multimers (capsomers), which, again, assemble into a stable virus capsid that can house the viral genome and associated components (e.g., nucleoproteins or enzymes). Thus, the assembled virus particles comprise many copies of capsid proteins and display a high (quasi-) symmetry that is often based on an icosahedron [35]. In icosahedral capsids, the particle architecture can be expressed in terms of the triangulation number. An icosahedron is a polyhedron consisting of 20 identical triangular faces that intersect at twelve vertices with five-fold rotational symmetry. Three-fold rotational axes are located in the center of each triangular face. The edges between the faces are intersected by two-fold rotational axes. The combination of these symmetry operations gives rise to the 532 point group of an icosahedron (Figure 3, lower right). In the context of an icosahedral virus particle, the triangulation number (T = h^2^ + hk + k^2^) can be interpreted as a measure of capsid size. It is calculated by the numbers of inter-capsomeric steps (iterated in h and k) that one has to traverse via (quasi-) six-fold symmetric capsomers, from one five-fold vertex to another.

The smallest and simplest virus capsids have an architecture corresponding to a triangulation number of T=1. In a T=1 capsid, each of the twelve five-fold symmetric vertices are occupied by a single capsid protein pentamer. Neighboring capsomers are pentamers that are also associated with the five-fold icosahedral vertices. No additional capsomers are present, which gives rise to a total number of 12 × 5 = 60 capsid proteins in the particle. To our knowledge, the only example of a T=1 sialic acid binding capsid is found in the Adeno-associated virus (AAV) of the *Parvoviridae* family. Sialic acid binding in AAV was reported to occur at the interface between two single capsid protein monomer chains, resulting in a total number of 60 binding sites in the capsid. The symmetry of the local binding site is defined by the shortest distance of a single binding site towards the rotational axis. The AAV capsid proteins that are responsible for sialic acid binding form pentamers. However, the binding sites themselves locate closer to the icosahedral three-fold symmetry axis than the five-fold capsomer/vertex axis. This implies that the 60 sialic acid binding sites of the AAV capsid are arranged in twenty local three-fold rather than twelve local five-fold symmetries [36].

**Figure 4:**
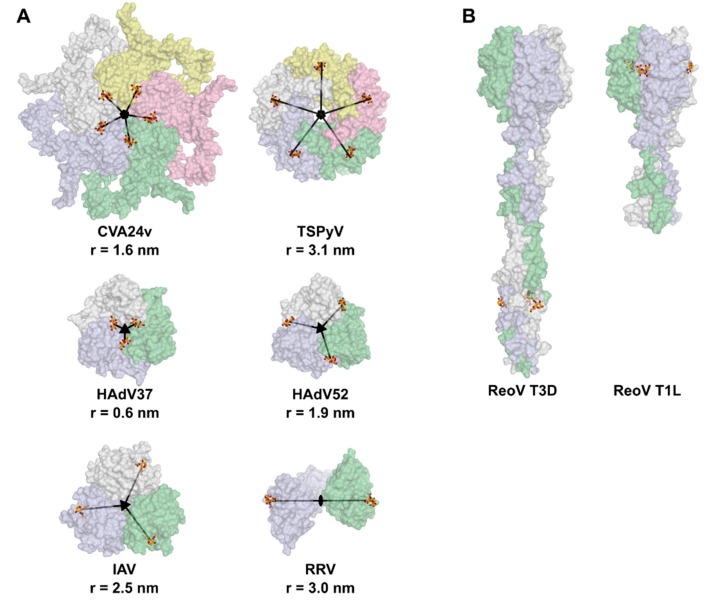
The symmetry relation of sialic acid binding sites in viral attachment factors. (**A**) Five-fold rotational binding site symmetry occurs in the attachment proteins of coxsackievirus A24 variant (CVA24v) and polyomaviruses, represented by the *Trichodysplasia spinulosa*-associated polyomavirus (TSPyV) VP1. Three-fold symmetry axes are found within the arrangement of sialic acid binding sites in human adenoviruses 37 (HAdV37) and 52 (HAdV52), as well as in the hemagglutinin of influenza-A virus (IAV). Rhesus rotavirus (RRV) lectins are dimers of a protruding VP4 domain with a two-fold sialic acid site rotational symmetry. The radii between the rotational axes and the binding sites are included. (**B**) In the human reoviruses (ReoV) of the type 3 (strain Dearing, T3D) and type 1 (strain Lang, T1L) fiber protein sigma1, the sialic acid sites display three-fold symmetry. However, their positions at the sides of the protein render them poorly useable for symmetric ligands, as exceedingly long linkers would be required to connect the sialic acid moieties. Single protomers of the attachment proteins are shown in distinct colors, respectively. PDB IDs 4q4x (coxsackievirus), 4u60 (polyomavirus), 1uxa (adenovirus 37), 6g47 (adenovirus 52), 3ubj (influenza A virus), 1kqr (rotavirus), 3s6x (reovirus T3D), and 4gu3 (reovirus T1L).

Virus particles comprising more than twelve capsomers must, by definition, have a higher triangulation number than T=1. *Picornaviridae* family members (such as coxsackieviruses, rhinoviruses, polioviruses, or enteroviruses) are viruses possessing three surface-exposed capsid proteins. The icosahedral five-fold penton positions are occupied by VP1 pentamers, which are bridged by one pseudo-hexon capsomer (VP2-VP3 heterohexamer), giving rise to pseudo-T=3 geometry (Figure 3). In the sialic acid-binding human coxsackievirus, A variant 24 (CVA24v), the binding site is located at the interface of two VP1 protomers, close to the five-fold icosahedral axis. VP2 and VP3 do not bind sialic acid, which results in a total of 60 binding sites, arranged in twelve local five-fold symmetries (Figure 4a) [37]. The distance between an individual sialic acid binding site and the local five-fold rotation axis of the VP1 pentamer amounts to ca. 1.6 nm.

In the members of both *Papillomaviridae* and *Polyomaviridae*, single capsid proteins named L1 and VP1, respectively, constitute the outer capsid. Both proteins exclusively form pentamers, which can occupy pentavalent and hexavalent positions in the mature particle, thus deviating from the quasi-equivalence principle proposed by Caspar and Klug in 1962 [35]. Here, a total number of 72 pentameric capsomers are arranged in a T=7d fashion, with a diameter of ca. 50 nm in the mature particles (Figure 3) [38,39,40]. The formation of smaller, non-viable lower-symmetry T=1 virus-like particles (VLPs) has also been described for both papillomaviruses and polyomaviruses [41,42,43]. Many members of the polyomavirus family bind sialic acid-based glycans using their VP1 proteins, so the binding sites on individual pentamers are always linked by local five-fold symmetry (Figure 4a, TSPyV). In all structures of polyomavirus-sialyl-oligosaccharide complexes, the majority of contacts between the protein and receptor can be attributed to sialic acid, with a small number of augmenting contacts to other saccharides providing specificity for a given glycan structure [27,44,45,46,47,48,49,50]. In contrast, papillomaviruses do not bind sialylated receptors, but instead interact with glycosaminoglycans to adhere to cells [51,52,53].

*Reoviridae* are a large family including, among others, the genera of orthoreoviruses and rotaviruses. Members of the *Reoviridae* family are double-shelled particles, in which the inner core layer consists of two core proteins arranged in a T=2* order and the outer capsid possesses T=13 geometry [54,55,56]. The diameter of mature virions is about 60–80 nm (Figure 3). In the case of mammalian reoviruses, the icosahedral vertex positions are occupied by the trimeric attachment protein sigma1, which markedly protrudes as a thin fiber from the virion surface. The sigma1 protein is about 50 nm long and has a head-and-tail morphology, with a globular head domain and an elongated tail that has flexible regions and partially inserts into the virion [57]. The location of sialic acid binding is type dependent. Type 1 reoviruses bind sialylated receptors in the protruding head domain while type 3 reoviruses use a binding site in a region near the mid-point of the tail (Figure 4b, ReoV T3D and T1L) [58,59]. Both sites primarily engage sialic acid with a small number of contacts, and in both cases, three-fold rotational symmetry is found between the binding sites within a single sigma1 trimer. Thus, 12 sigma1 trimers give rise to 36 sialic acid binding sites in orthoreoviruses.

Rotaviruses also have protruding domains that recognize carbohydrate receptors, which are also dimers of the virus protein 4 (VP4) [60]. While some of the more pathogenic human viruses bind HBGAs, animal rotaviruses primarily engage sialic acid-based receptors [61,62,63]. Each rotavirus VP4 subunit carries a single sialic acid binding site, which is related to the two-fold rotational symmetry in the dimer (Figure 4a, RRV) [64]. In further contrast to orthoreoviruses, these fibers do not coincide with the five-fold vertices but rather associate at the margins of the five capsomers around the five-fold vertices, resulting in a total number of 60 VP4 dimers and 120 sialic acid binding sites [65].

*Adenoviridae* members are large viruses with a diameter of about 90 nm and a T=25 icosahedral capsid (Figure 3) [66,67]. They display trimeric fibrous attachment proteins, known as the fiber, at their icosahedral vertices. Similar to the reovirus sigma1, the adenovirus fiber has a head-and-tail morphology and features a globular head domain (the knob) that projects from the virus surface, and a tail (the shaft) that inserts into the virus particle [67]. The sialic acid binding sites of the different structurally characterized adenovirus types are located in the fiber knob domain [26,68]. While these sites differ in location in different adenoviruses, they are all linked by three-fold symmetry and lie in close proximity to each other (Figure 4a, HAdV37 and HAdV52).

Although this review focuses on non-enveloped viruses, it should still be mentioned that some of the concepts described above also apply to enveloped viruses, such as coronaviruses, paramyxoviruses, and orthomyxoviruses. While global symmetry measures for these viruses are elusive (since their attachment proteins are membrane-bound, somewhat mobile, and do not follow easily-appreciated assembly rules), the existing local binding site symmetries within multimeric attachment proteins can still be exploited for rational multivalent inhibitor design. A prominent example is the influenza A virus hemagglutinin, which is a homotrimeric protein bearing three individual sialic acid binding sites [69]. These binding sites are related by local three-fold rotational symmetry, with a distance of 2.5 nm from the three-fold axis (Figure 4a, IAV).

## 3. Sialic Acid-Based Design of Anti-Viral Compounds

### 3.1. Monovalency

The sialic acid moieties of glycosylated proteins and/or glycolipids are required for the attachment, entry, and productive infection of many viruses. In the majority of cases, the viral binding sites are surface-exposed and engage terminal sialic acid residues with a range of hydrophilic and hydrophobic contacts. The available structural data for receptor–ligand interactions at an atomic resolution can inform the synthesis of high affinity inhibitors via the chemical modifications of sialic acids. A prominent example of this approach is the inhibition of influenza virus neuraminidases. These enzymes recognize and hydrolyze terminal sialic acids of cell surface glycans and are vital for the release of viral progeny [70,71]. An early non-selective prototype inhibitor of neuraminidases was the sialic acid derivate 2-deoxy-2,3-didehydro-*N*-acetylneuraminic acid (Neu5Ac2en, DANA) [72,73]. DANA can engage and block the binding sites of neuraminidase of both ortho- and paramyxoviruses, thereby suppressing the activity of these enzymes. In the early 1990s, Mark von Itzstein and colleagues made substantial advances towards selective and highly affine influenza A virus inhibitors. They discovered that the derivatization of the 4-*O*-hydroxyl group of DANA with a guanidine moiety drastically increases affinity to the influenza A virus neuraminidase, which resulted in the anti-influenza drug Zanamivir [74,75]. Subsequently, the structures of the planar sialic acid transition state during the neuraminidase reaction became available. The analogous compounds of sialic acid’s transition state based on benzoic and shikimic acids, and subsequently the drug Oseltamivir [76,77,78,79], benefit from the increased affinity to neuraminidase compared to the native Neu5Ac structure [80,81]. Unfortunately, the family of compounds related to Zanamivir or Oseltamivir do not act against viruses that do not possess neuraminidase activity but instead engage undistorted sialic acids via a hemagglutinin (e.g., many non-enveloped viruses discussed in chapter 2 of this review). In cases where affinities between sialylated glycans and hemagglutinins have been measured, the interactions were shown to have dissociation constants in the millimolar range [27,28,29,30]. The firm cell attachment of these viruses is usually driven by high avidity, relying on a large number of identical binding sites that can engage receptors.

### 3.2. Polyvalency

In terms of the inhibition of attachment, monovalent sialic acid analogues are generally poor choices, as they are not able to engage a single binding site in a viral attachment protein with high affinity. Therefore, polyvalent sialic acid-based compounds can sometimes be more effective. In parallel to the development of monovalent sialic acid transition state analogues against the influenza virus neuraminidases, polymeric or nanoparticle based sialic acid conjugates were developed to also prohibit influenza virus infection by extensive binding to the viral hemagglutinin, thereby shielding the virus particle. The influenza virus hemagglutinin can be blocked by multivalent sialosides that vary in chemical composition, size, branching complexity, and ligand density [82,83,84,85,86]. Particularly the effects of the nature of the scaffold (or platform) and spatial sialic acid distribution in polyvalent viral attachment inhibitors are still being investigated [87]. Recently, excellent reviews covering the composition and biophysical properties of multivalent sialosides were published by Bhatia et al. [88] and Lu et al. [89], so we will not discuss this topic further here.

### 3.3. Oligovalency

Next to the design of monovalent inhibitors or highly complex polyvalent macromolecular structures, a third and rather minimalistic design strategy makes use of the internal symmetry of the addressed targets. As elucidated earlier in this review, many viral attachment proteins employ a local symmetry of their receptor binding sites that represent a convenient framework for the rational design of small, tailored inhibitors. In contrast to monovalent inhibitors, oligovalent compounds can benefit from cooperative binding or avidity. Nevertheless, they are small enough for nephric clearance and display a higher bioavailability than most macromolecular polyvalent structures [90]. The design of oligovalent inhibitors is usually based on available crystal structures, taking the symmetry and topology of the target into account. The accessibility of the individual ligand binding sites and their distances from an eventual symmetry axis should be considered. For binding sites in close proximity to the local symmetry axis, it can be feasible to start from a central molecule, which can be derivatized by ligand terminating linkers, resulting in an inhibitor with a radial structure. For binding sites that are far from the local symmetry axis or in a recessed part of the target protein, it may be viable to directly link ligand molecules to each other instead of using a central scaffold. Direct linking of ligand molecules may eventually result in the design of a circular oligovalent inhibitor. So far, structural information on attachment proteins bound by oligovalent inhibitors is scarce. X-ray crystallography analysis is usually hampered by the high flexibility of spacer groups and scaffolds. These non-binding parts of the inhibitors typically do not assume defined conformations, resulting in weak and uninterpretable electron density. This was the case in the study of the Shiga-like toxin inhibitor STARFISH, described in the introduction of this review, where interpretable electron density was only present for the bound ligand moieties of the compound. Still, the structural characterization of virus-inhibitor interactions in order to design or optimize anti-viral compounds has been the subject of extensive research.

Recently, a research paper by Lu et al. [91] reported on the trivalent design of sialic acid bearing inhibitors against influenza A virus hemagglutinin, showing a greater than 400-fold increase in affinity compared to the monovalent ligand. However, structural data of the interactions between the inhibitor and hemagglutinin were not reported in that study.

**Figure 5:**
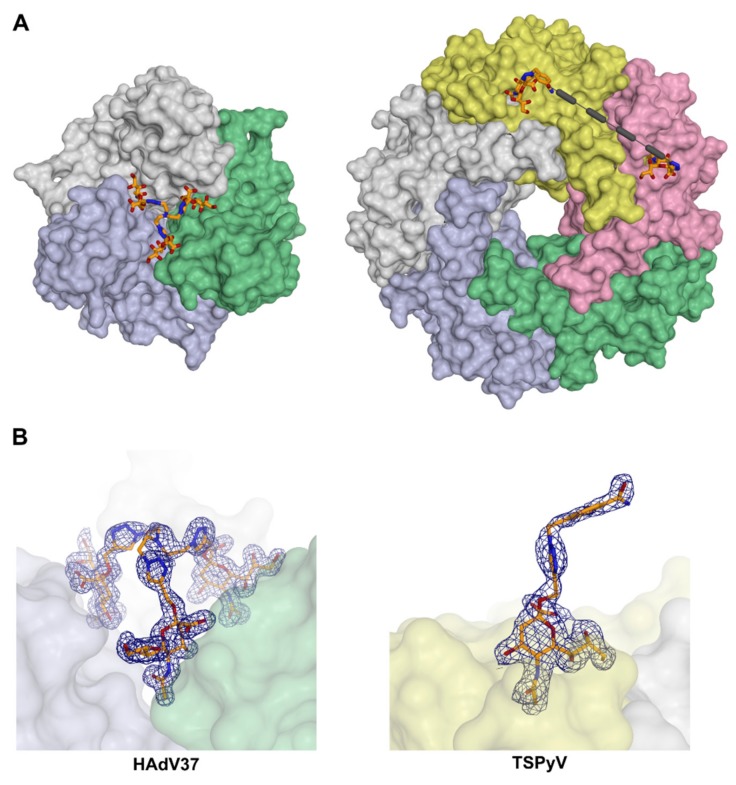
Sialic acid based anti-viral compounds. (**A**) Structure of the HAd37 fiber knob in a complex with a trivalent scaffold functionalized with sialic acid (**left**) and the structure of the *Trichodysplasia spinulosa*-associated Polyomavirus (TSPyV) VP1 pentamer in a complex with a divalent sialic acid-presenting compound (**right**). For simplification, the compound is only depicted in two of the five binding sites of the TSPyV VP1, although it is present in the other three, as well. Absent parts of the ligand backbone structure are represented with dashed lines. (**B**) Corresponding electron densities for the ligands shown above. Proteins are depicted as surfaces with distinctly colored single protomers and organic compounds in stick representation, respectively. The electron density 2Fo-Fc maps are represented as blue meshes and displayed at a level of 1 σ (standard deviation of the mean) and a radius of 1.6 Å around the respective ligand. PDB IDs 4xqa (adenovirus 37) and 6hkv (polyomavirus).

One study of a central group-based anti-viral inhibitor, which contains structural data, can be found for adenoviruses. *Adenoviridae* members carry trimeric fibers terminating in the knob domain, which engages sialic acid-based receptors in some adenoviruses. Human Adenovirus 37 (HAdV37), which causes Epidemic Keratoconjunctivitis (EKC), carries three individual binding sites for sialic acids in its fiber knob, which are located at a distance of 6 Å from the local three-fold symmetric axis (Figure 4a) [68]. The physiologic receptor of HAdV37 is the glycan portion of ganglioside GD1a. This glycan is a branched hexasaccharide with two terminal sialic acid moieties, both of which were shown to simultaneously occupy two of the three available sialic acid binding sites in the same fiber knob [92]. Based on this observation, Spjut et al. (2011) [93] designed and synthesized a symmetric, tridentate sialylated inhibitor, which is capable of occupying all three binding sites of the fiber knob at once. The first generation of these inhibitors was designed around a central tris(2-aminoethyl) amine group. It utilized flexible spacers between the central group and the sialic acid ligand, in order to minimize the chance of steric hindrance of the inhibitor docking. The resulting compounds demonstrated a potency increase of four orders of magnitude compared to monomeric sialic acid [93]. Recently, in the second-generation inhibitors, the binding affinity could be improved even further by using a shorter, triazole-based linker structure, which was based on the results of the first-generation complex structures. The more compact and rigid design resulted in an additional 140-fold increase in potency [29]. The crystal structures of the second-generation inhibitor molecules in a complex with adenovirus fiber knob proteins verify the trivalent binding mode by also displaying the electron density of the linkers (Figure 5a,b, left).

An example of directly-linked ligand moieties was shown in a study of potential polyomavirus inhibitors, in which Baier et al. [94] synthesized so-called divalent sialylated glycooligopeptides. They solved the structures of two glycooligopeptide compounds in a complex with the major capsid protein (VP1) pentamer of the *Trichodysplasia spinulosa*-associated polyomavirus (TSPyV), which is associated with abnormal skin growth in immunocompromised patients. The VP1 pentamer carries five individual sialic acid binding sites at a distance of 35 Å between neighboring sites [50,94]. The glycooligopeptide-VP1 complex structures displayed a similar ligand binding mode that was reported for sialic acid in an earlier study [50] and showed, for the compounds, that the linker between the ligand and the scaffold occupies the space that is usually targeted by the natural glycan receptor moieties (Figure 5a,b, right). However, the interconnectivity of functional receptors by the scaffold remained undetermined (probably due to their flexibility) and are, therefore, the average of several potential bridging modes on top of the pentamers [94].

## 4. Conclusions

Many viral lectins or attachment proteins rely on the recognition of sialic acids. Due to the high symmetry of viral particles and the occurrence of local symmetry within commonly multimeric viral proteins, sialic acid binding often occurs in a symmetrical context, too. This symmetry is a convenient framework for the design of tailor-made inhibitory ligands competing with the high avidity of virus–cell interactions. Structural biology techniques, such as X-ray crystallography and single-particle electron cryo-microscopy (cryo-EM), can now tackle the visualization of viral attachment and carbohydrate interactions with unprecedented scope and detail. The resulting structural data can be used for the optimization of anti-viral compounds, which could be developed further into high-affinity drug candidates.

However, challenges in compound design remain. For example, the higher rigidity of a multivalent ligand does not necessarily translate into improved binding. In the case of HAd37, a rigid compound bound 200-fold less well to the fiber knob than a related compound that had higher flexibility [29]. This suggests that the perfect positioning of all sialic acids in the binding site, especially for larger inhibitor molecules, is difficult to achieve, and a certain degree of flexibility might help with the high-affinity binding of the inhibitor.

Another limiting factor for oligovalent inhibitors is the positioning of the binding pockets. In the case of the trivalent adenovirus inhibitor, the binding sites are on the very top of the knob domain, so there is enough space for linkers and a central core (Figure 4a). In contrast, the sialic acid binding sites of the reovirus sigma 1 fibers are located on the side of the protein (Figure 4b), which makes it challenging to design an appropriate oligovalent inhibitor. Additional challenges are the long-term stability, convenient synthesis, and, for later application, reasonable bioavailability of multivalent compounds.
